# Testing peripheral and central therapies for provoked vestibulodynia: results from the UPDATe trial

**DOI:** 10.1080/07853890.2026.2732293

**Published:** 2026-10-10

**Authors:** Chloe Shudt, Erin T. Carey, Elizabeth J. Geller, Andrea Rapkin, Debbie Farb, Haley Cutting, Jasmyn Akaninwor, Jesse DeLaRosa, Christopher Stirling, Andrey Bortsov, Pearl Rowe, Sheng Luo, Marguerita E. Klein, Andrea G. Nackley

**Affiliations:** a Center for Translational Pain Medicine, Department of Anesthesiology, Duke University School of Medicine, Durham, NC, USA; b Department of Obstetrics and Gynecology, University of North Carolina at Chapel Hill, Chapel Hill, NC, USA; c Department of Obstetrics and Gynecology, University of California at Los Angeles, Los Angeles, CA, USA; d Duke Clinical Research Institute, Duke University, Durham, NC, USA; e Department of Biostatistics and Bioinformatics, Duke University, Durham, NC, USA; f Department of Pharmacology and Cancer Biology, Duke University School of Medicine, Durham, NC, USA

**Keywords:** Chronic primary pain, nociplastic pain, nortriptyline, estradiol, lidocaine, pelvic pain

## Abstract

**Introduction:**

Vestibulodynia (VBD) is a common chronic pelvic pain condition with heterogeneous clinical presentation, including subtypes distinguished by the presence (VBD-central; VBD-c) or absence (VBD-peripheral; VBD-p) of comorbid pain conditions. Despite frequent prescribing of topical therapies such as lidocaine/estradiol and centrally-acting agents such as tricyclic antidepressants, high-quality evidence for efficacy, alone or in combination, remains limited. This study evaluated effects of peripheral, central, and combined pharmacologic treatments for two VBD subtypes and whether treatment response varied by subtype.

**Patients and Methods:**

We conducted a multicenter, randomized, double-blind, placebo-controlled, 2 × 2 factorial trial (ClinicalTrials.gov identifier: NCT03844412) at two academic medical centers. Women aged 18–50 with clinician-confirmed VBD were randomized to one of four treatment arms: 1) topical 5% lidocaine + 0.05% estradiol vaginal cream and placebo pill, 2) oral nortriptyline pill and placebo cream, 3) combined active pill and cream, or 4) double placebo for 16 weeks. The primary outcome was provoked vulvovaginal pain by a standardized tampon test (0–10 numeric rating scale).

**Results:**

Of 209 participants, 69 had VBD-p and 140 had VBD-c. Tampon test pain decreased modestly across all arms, with the greatest proportion of decrease in the combined nortriptyline plus lidocaine/estradiol group, but there was no difference between subtypes. In the primary model, baseline pain strongly predicted Week 16 pain (β = 0.68, *p* < 0.001), and while many participants had a decrease in pain over time, no treatment produced a statistically significant reduction compared with placebo.

**Conclusions:**

In this trial, neither peripheral nor centrally targeted pharmacotherapies demonstrated superiority over placebo in reducing provoked vulvovaginal pain at 16 weeks.

## KEY MESSAGES

1. In this multicenter, randomized, placebo-controlled factorial trial, neither oral nortriptyline nor topical lidocaine/estradiol, alone or in combination, demonstrated superiority over placebo in reducing provoked vulvovaginal pain at 16 weeks.
2. Despite the absence of significant treatment effects, 33.3% to 61.3% of women in the treatment-compliant population experienced pain improvement across all study arms, highlighting substantial nonspecific and individual-level responses.
3. Heterogeneous, subtype-specific response patterns and baseline differences in psychosocial and functional burden underscore the need for precision-based, phenotype-informed approaches to vestibulodynia treatment.

## Introduction

Vestibulodynia (VBD) is a chronic primary pain condition (CPPC) characterized by recurrent pain of the vulvar vestibule at the vaginal opening for at least three months [1,2]. Beyond physical discomfort, VBD is associated with reduced quality of life across multiple psychosocial domains, and has an estimated annual economic burden of $31 to $72 billion in the United States alone [3,4]. Clinical presentation is heterogeneous, varying in onset, spontaneity, pain quality, and temporal pattern, reflecting underlying biological complexity [5–8]. Proposed mechanisms span chronic inflammation and immune dysregulation [9–15], peripheral neural hyperinnervation and nociceptor sensitization [16–20], central sensitization and altered pain processing [21–24], hormonal and epithelial factors [25–28], urogenital infection [15,29,30], pelvic floor muscle abnormalities [31–33], and psychosocial contributors [34–38].

This heterogeneity in clinical presentation has motivated the development of clinical subtyping frameworks intended to capture mechanistic differences and inform treatment selection [5,8,39–43]. One subtyping approach categorizes individuals by the presence or absence of comorbid CPPCs, such as migraine, fibromyalgia, irritable bowel syndrome, bladder pain syndrome, and temporomandibular disorder, based on evidence supporting shared biological pathways and heightened multisite pain vulnerability [10,39,44–48].

Management of VBD commonly includes a range of treatments [49,50], including those directly targeting the vestibular mucosa [51–54], pelvic floor musculature [55–59], or central pain pathways [60–62]. Frequently prescribed local therapies include topical anesthetics, such as lidocaine, and hormonal treatments, such as topical estrogen, which aim to reduce peripheral nerve excitability, restore tissue integrity, and mitigate local inflammatory signaling [63,64]. Centrally acting agents, including antidepressants and anticonvulsants, such as nortriptyline, are used to modulate altered pain processing through effects on descending inhibitory pathways and neuroinflammatory signaling [14,65,66]. Although multidisciplinary approaches have been proposed, rigorous evidence demonstrating superiority over placebo or usual care remains limited [67–69].

Despite the high prevalence of VBD and the routine use of both local and centrally acting pharmacologic treatments, a critical gap remains in evaluating their efficacy across clinically meaningful subtypes. Prior trials rarely stratified participants by pain subtype and often excluded individuals with comorbid chronic primary pain conditions [50], limiting the ability to determine whether treatment response differs by underlying mechanisms.

The vestibulodynia: understanding pathophysiology and determining appropriate treatments (vestibulodynia: UPDATe) study addressed this gap by examining pharmacologic treatment effects according to VBD-c and VBD-p subtype. We hypothesized that women with VBD-c would derive greater benefit from centrally acting treatment, whereas individuals with VBD-p would preferentially respond to local pharmacologic treatment.

## Patients and methods

### Study design

The Vestibulodynia UPDATe trial is a multicenter, double-blind, placebo-controlled, randomized trial employing a two-by-two factorial design (ClinicalTrials.gov: NCT03844412). The study evaluates the efficacy of two treatments (topical lidocaine/estradiol compound cream and oral nortriptyline) administered alone, in combination, or compared to matched placebos. Participants were randomized to one of four treatment groups: 1) lidocaine/estradiol compound cream and oral placebo pill, 2) placebo cream and oral nortriptyline pill, 3) lidocaine/estradiol compound cream and oral nortriptyline pill; or 4) placebo cream and placebo pill. This 16-week treatment phase was followed by outcome assessments at baseline and weeks 8, 16, and 24 with the primary endpoint defined as change in tampon test pain at 16 weeks, as longitudinal SF-MPQ data were unusable (see below).

Recruitment occurred between 4 November 2019 and 1 December 2023 at two sites: the University of North Carolina at Chapel Hill and the University of California, Los Angeles, with ethical approval obtained by the Duke University Institutional Review Board protocol (Pro00100678). This analysis includes 209 cisgender women with vestibulodynia confirmed by study clinicians at enrollment. All research was performed in accordance with the principles stated in the Declaration of Helsinki and oversight provided by a Data Safety Monitoring Board. We report our study in accordance with the CONSORT 2025 statement, which provides a minimum set of items to improve the transparency and completeness of reporting in randomized trials (Supplement A) [70].

### Inclusion and exclusion criteria

Women aged 18–50 years with clinician-confirmed vestibulodynia were eligible if they reported vulvar pain for at least three months, rated pain severity ≥ 3 on a 0–10 numeric rating scale (NRS) with insertional sexual activity and/or with touch to the vaginal opening, and demonstrated pain ≥ 3 on standardized tampon test at enrollment.

Key exclusion criteria included active vulvar or vaginal infection, dermatoses, or dermatitis, untreated symptomatic vaginal atrophy, prior vestibulectomy, pregnancy, breastfeeding, postmenopausal status, uncontrolled medical or psychiatric conditions, intolerance or contraindications to study medications, recent use of medications or interventions that could confound pain outcomes and generalized vulvodynia without vestibulodynia. Complete inclusion and exclusion criteria are detailed in the published study protocol [71] and Supplemental Table 1.

### Recruitment, screening, and enrollment

Primary recruitment occurred through subspecialty pelvic pain clinics and gynecologic clinics at both sites. Additional strategies included flyer distribution, social media outreach (e.g. Instagram, Facebook, Twitter/X), study-specific website (sites.duke.edu/updatestudy), mass emails to UNC and UCLA university communities and electronic health record queries using vestibulodynia-associated ICD-9 or ICD-10 codes. Information was also posted on clinicaltrials.gov and other websites (e.g. Duke List, National Vulvodynia Association).

Screening was conducted *via* telephone call, email, or in-person clinic visits to assess preliminary eligibility. Potentially eligible participants were invited to attend an in-person screening visit (Visit 1). After providing written informed consent, participants completed urine pregnancy testing and a standardized tampon test, then rated their current pain with vaginal penetration during sexual activity and with touch to the vaginal opening using a 0–10 NRS. All inclusion and exclusion criteria were then reviewed in person with study staff. Participants who met all eligibility criteria, had a negative pregnancy test, and rated pain as ≥ 3/10 on the tampon test, and with either vaginal penetration or vestibular touch were immediately enrolled and proceeded to complete baseline data collection during the same visit.

### Standardized tampon test

Participants were given the same tampon: Original Regular Tampax^™^, in a standard cardboard applicator. The tampon itself was approximately 5.5 cm long × 1.5 cm diameter, with a 12.8-cm cardboard applicator. The participant was instructed to: 1) not lubricate the tampon, 2) using only the supplied cardboard applicator, insert the tampon and deposit it fully into the vagina above the level of the hymenal ring, 3) withdraw the applicator, 4) immediately remove the tampon by pulling on the tampon string, and 5) rate the pain experienced during the entire insertion-and-removal procedure on a 0–10 NRS, where 0 = no pain and 10 = worst possible pain.

### Study medications

Participants were randomly assigned to one of four treatment combinations: (1) lidocaine 5% + 0.05% estradiol compound cream and oral placebo, (2) placebo cream and oral nortriptyline (titrated up to 50 mg or highest tolerated dose), (3) lidocaine 5% + 0.5% estradiol compound cream and oral nortriptyline; or (4) placebo cream and oral placebo. Medication adherence was tracked *via* weekly email prompts to enter usage data into Research Electronic Data Capture (REDCap) calendar.

### Randomization and blinding

The Duke Clinical Research Institute (DCRI) generated randomization sequences using SAS© (Cary, NC) with permuted blocks stratified by enrollment site. Assignments were loaded into REDCap and concealed from participants and study personnel. Matching placebo cream and capsules, identical in appearance to the active study medications, maintained blinding integrity.

### Assessment of outcome measures

Study assessments occurred at four time points: baseline (week 0), mid-treatment (week 8), end-of-treatment (week 16), and post-treatment follow-up (week 24).

The original co-primary outcomes were (1) change in provoked vulvovaginal pain measured by standardized tampon test and (2) change in self-reported pain measures assessed *via* the Short-Form McGill Pain Questionnaire (SF-MPQ) [72,73]. However, due to an inadvertent change in REDCap settings which led to incomplete longitudinal collection of SF-MPQ data during the trial, SF-MPQ outcomes were not available at follow-up time points for the majority of participants. Accordingly, the present analysis focuses on the tampon test outcome. Secondary outcomes included quantitative sensory testing performed at the same study visits. A complete assessment schedule is published in the study protocol [71].

### VBD subtype designation

Participants were classified into two subtypes based on the presence or absence of comorbid chronic primary pain conditions (CPPCs). Those with vestibulodynia alone were designated peripheral vestibulodynia (VBD-p), while those with vestibulodynia and ≥1 CPPC were designated as centralized vestibulodynia (VBD-c). Comorbid pain conditions were assessed *via* standardized self-report questionnaire evaluating nine chronic pain syndromes: fibromyalgia, temporomandibular disorder, back pain, irritable bowel syndrome, chronic tension-type headache, chronic migraine, chronic fatigue syndrome, endometriosis, and bladder pain syndrome/interstitial cystitis.

### Statistical analysis

Baseline characteristics were compared between VBD subtypes using t-tests or Wilcoxon rank-sum tests for continuous variables and chi-square or Fisher’s exact tests for categorical variables.

The primary efficacy analysis employed an analysis of covariance (ANCOVA) framework, with week 16 tampon test pain as the dependent variable and baseline tampon test pain as a covariate. The prespecified model included main effects for oral/pill treatment, cream treatment, and their interaction to evaluate the factorial treatment design while adjusting for baseline pain severity. Study site was included as a covariate to account for stratified randomization.

Secondary analyses evaluated treatment effect modification by VBD subtype through inclusion of subtype as a main effect and subtype-by-treatment interaction terms. Sensitivity analyses were conducted in the treatment-compliant population (defined by DCRI adherence criteria) using both unadjusted change scores and ANCOVA models analogous to the primary analyses.

Analyses were conducted in a modified intent-to-treat population, unless otherwise specified, including all randomized participants with available baseline and Week 16 tampon test pain measurements. The number of participants included in each analysis is reported in the corresponding tables and summarized in Figure 1.

**Figure 1.**
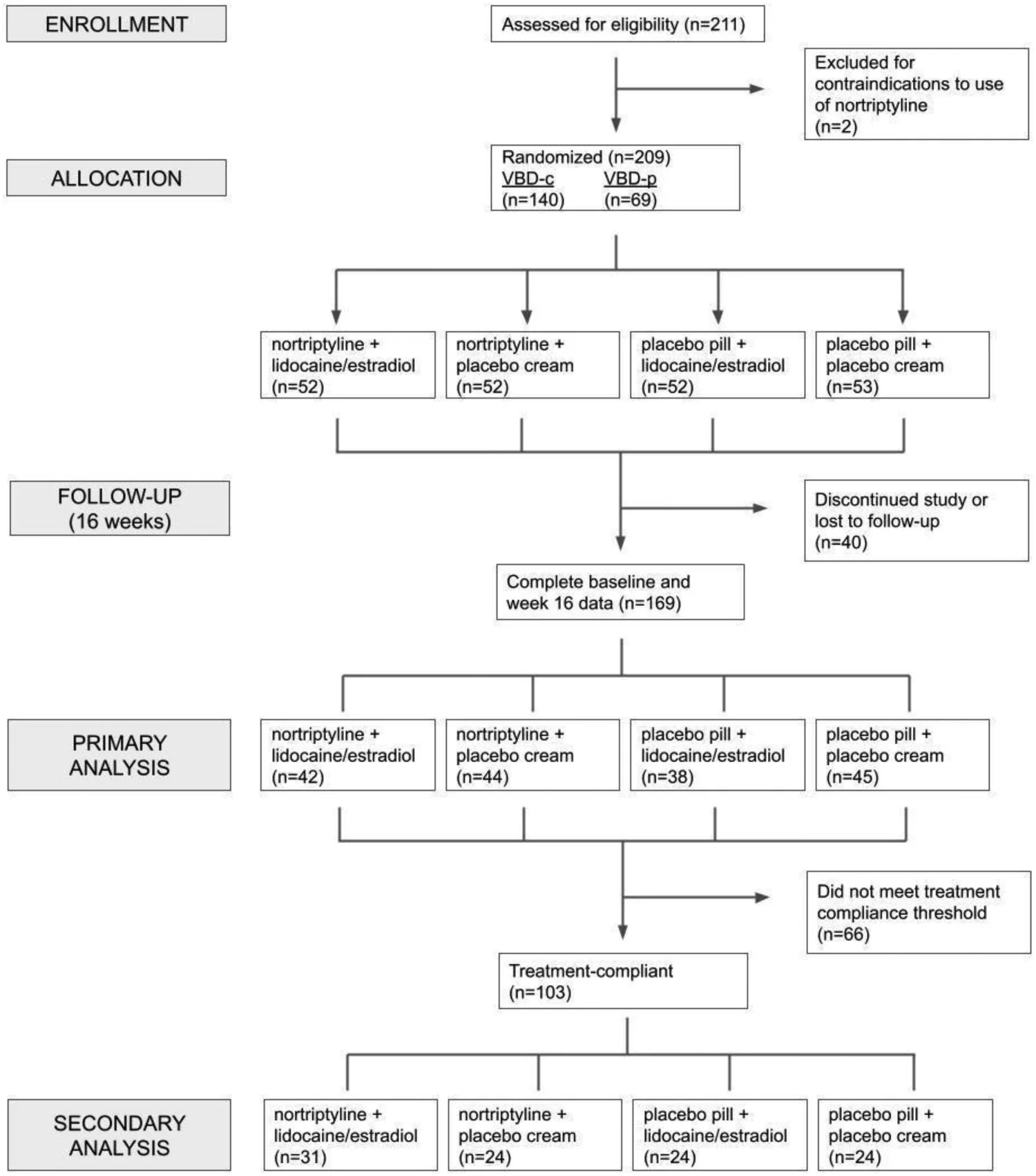
CONSORT flow diagram.

Statistical significance was assessed using two-sided tests with α = 0.05. Analyses and graphing were performed using Python (Python Software Foundation, Wilmington, DE, USA).

## Results

### Participant characteristics

Of 209 enrolled (111 UNC, 98 UCLA), 140 (67%) participants were classified as VBD-c and 69 (33%) as VBD-p (Figure 1). Baseline demographic characteristics were generally well-balanced between subtypes with respect to age, race, and ethnicity, employment status, household income, insurance coverage, and marital status (Table 1).

**Table 1.** Demographic characteristics.

Characteristic	Overall (*N* = 209)	VBD-c (*N* = 140)	VBD-p (*N* = 69)
**Age** (years ± SD)	26.3 ± 7.1	27.1 ± 7.4	24.7 ± 6.3
**Ethnicity**			
Non-Hispanic or Latino	182 (87.1%)	123 (87.9%)	59 (85.5%)
Hispanic or Latino	27 (12.9%)	17 (12.1%)	10 (14.5%)
**Race (all that apply)**			
White	157 (75.1%)	104 (74.3%)	53 (76.8%)
Asian	49 (23.4%)	28 (20.0%)	21 (30.4%)
Black or African American	22 (10.5%)	18 (12.9%)	4 (5.8%)
American Indian/Alaska Native	4 (1.9%)	3 (2.1%)	1 (1.4%)
Native Hawaiian or Other Pacific Islander	2 (1.0%)	2 (1.4%)	0 (0.0%)
**Employment status**			
Full-time employed	93 (44.5%)	62 (44.3%)	31 (44.9%)
Part-time employed	65 (31.1%)	47 (33.6%)	18 (26.1%)
Unemployed	51 (24.4%)	31 (22.1%)	20 (29.0%)
**Education level**			
Started or completed postgraduate course work	57 (27.3%)	37 (26.4%)	20 (29.0%)
Graduated from college	72 (34.4%)	52 (37.1%)	20 (29.0%)
Some college	68 (32.5%)	43 (30.7%)	25 (36.2%)
High school or less	12 (5.8%)	8 (5.7%)	4 (5.8%)
**Household income**			
More than $100,000	69 (33.0%)	51 (36.4%)	18 (26.1%)
$50,000 to $100,000	54 (25.8%)	38 (27.1%)	16 (23.2%)
$0 to $50,000	62 (29.7%)	36 (25.7%)	26 (36.2%)
Decline to answer	24 (11.5%)	15 (10.7%)	9 (13.0%)
**Insurance (all that apply)**			
Private	166 (79.4%)	110 (78.6%)	56 (81.2%)
Other	32 (15.3%)	24 (17.1%)	8 (11.6%)
Medicare or Medicaid	12 (5.7%)	6 (4.3%)	6 (8.7%)
Uninsured	1 (0.5%)	1 (0.7%)	0 (0.0%)
**Marital status**			
Single	115 (55.0%)	74 (52.9%)	41 (59.4%)
Married or living with significant other	91 (43.5%)	63 (45.0%)	28 (40.6%)
Divorced/separated	3 (1.4%)	3 (2.1%)	0 (0.0%)

### Gynecologic history

Participants with VBD-c reported significantly higher rates of pre-menstrual syndrome (OR 2.19, 95% CI 1.20–4.00) and painful menstrual cycles (OR 2.65, 95% CI 1.45–4.85) compared with VBD-p (Table 2). No significant differences were observed between subtypes with respect to history of pre-menstrual dysphoric disorder, recurrent vaginal infections, sexually transmitted infections, or current contraceptive use (Supplemental Table 2). In the past 30 days, 169 of 209 participants (80.9%) reported sexual activity; among the 192 participants with a history of vaginal intercourse, 191 (99.5%) reported pain during intercourse.

**Table 2.** Gynecologic history.

Characteristic	Overall (*N* = 209)	VBD-c (*N* = 140)	VBD-p (*N* = 69)	OR (95% CI)	p-value
**History of pre-menstrual syndrome (PMS)**				2.19 (1.2 to 4.0)	0.0157
No	69 (33.0%)	38 (27.1%)	31 (44.9%)		
Yes	140 (67.0%)	102 (72.9%)	38 (55.1%)		
**PMS severity**	(*N* = 140)	(N=			
Mild	79 (56.4%)	54 (52.9%)	25 (65.8%)		
Moderate	51 (36.4%)	39 (38.2%)	12 (31.6%)		
Severe	10 (7.1%)	9 (8.8%)	1 (2.6%)		
**History of pre-menstrual dysphoric disorder (PMDD)**				0.57 (0.17 to 1.95)	0.5108
No	198 (94.7%)	134 (95.7%)	64 (92.8%)		
Yes	11 (5.3%)	6 (4.3%)	5 (7.2%)		
**Painful menstrual cycles**				2.65 (1.45 to 4.85)	0.0024
No	69 (33.0%)	36 (25.7%)	33 (47.8%)		
Yes	140 (67.0%)	104 (74.3%)	36 (52.2%)		
Pain severity during menstrual cycles					
Mild	34 (24.3%)	23 (22.1%)	11 (30.6%)		
Moderate	72 (51.4%)	50 (48.1%)	22 (61.1%)		
Severe	34 (24.3%)	31 (29.8%)	3 (8.3%)		
**History of recurrent vaginal infections**				1.18 (0.61 to 2.29)	0.7428
No	153 (73.2%)	101 (72.1%)	52 (75.4%)		
Yes	56 (26.8%)	39 (27.9%)	17 (24.6%)		
Type of vaginal infection (all that apply)					
Yeast Infection	50 (89.3%)	36 (92.3%)	14 (82.4%)		
Bacterial Vaginosis	33 (58.9%)	23 (59.0%)	10 (58.8%)		
**History of vaginal intercourse**				1.12 (0.4 to 3.16)	0.99
No	17 (8.1%)	11 (7.9%)	6 (8.7%)		
Yes	192 (91.9%)	129 (92.1%)	63 (91.3%)		
Pain during vaginal intercourse					
No	1 (0.5%)	1 (0.8%)	0 (0.0%)		
Yes	191 (99.5%)	128 (99.2%)	63 (100.0%)		
**History of an STD**				1.45 (0.55 to 3.86)	0.6073
No	186 (89.0%)	123 (87.9%)	63 (91.3%)		
Yes	23 (11.0%)	17 (12.1%)	6 (8.7%)		
**Current birth control use**				1.3 (0.69 to 2.45)	0.5089
No	59 (28.2%)	37 (26.4%)	22 (31.9%)		
Yes	150 (71.8%)	103 (73.6%)	47 (68.1%)		
**History of hormonal birth control use**				1.64 (0.88 to 3.05)	0.1582
No	61 (29.2%)	36 (25.7%)	25 (36.2%)		
Yes	148 (70.8%)	104 (74.3%)	44 (63.8%)		
**Current hormonal treatment for pre-menopausal symptoms**					
No	133 (89.9%)	96 (92.3%)	37 (84.1%)		
Yes	15 (10.1%)	8 (7.7%)	7 (15.9%)		

### Baseline pain and functional impact

Pain intensity measures (tampon insertion pain, average vulvar pain over prior 2 weeks) did not differ between VBD-c and VBD-p (Table 3). However, VBD-c participants reported significantly greater pain-related functional interference, including impaired work ability (OR 6.57, 95% CI 2.20–19.66) and difficulty falling asleep (OR 3.15, 95% CI 1.54–6.44), in both unadjusted and adjusted models controlling for age, ethnicity and recruitment site.

**Table 3.** Baseline vulvovaginal pain intensity and pain interference.

Outcome	Subtype	Mean	Median (IQR)	p-value	Adjusted OR (95% CI)	Adjusted p-value
Pain with tampon insertion	VBD-c	4.13	4 (3–6)			
	VBD-p	4.10	4 (3–6)	0.9667	1.03 (0.64 to 1.81)	0.7671
Average vulvar/genital pain (past 2 weeks)	VBD-c	4.50	4 (3–6.25)			
	VBD-p	4.67	5 (3–7)	0.5772	0.86 (0.51 to 1.44)	0.5677
Vulvar/vaginal pain interference with ability to work (past 3 mo)	VBD-c	0.49	0 (0–1)			
	VBD-p	0.10	0 (0-0)	0.0004	6.57 (2.20 to 19.66)	0.0008
Vulvar/vaginal pain interference with ability to fall asleep (past 3 mo)	VBD-c	0.79	0 (0–1)			
	VBD-p	0.23	0 (0-0)	0.0003	3.15 (1.54 to 6.44)	0.0016

Adjusted analyses control for age, ethnicity, and recruitment site.

### Baseline psychological and somatic measures

VBD-c participants demonstrated significantly higher somatic awareness (Pennebaker Inventory of Limbic Languidness (PILL) adjusted mean difference 14.85, 95% CI 9.11–20.59; *p* < 0.0001). Psychological distress, as measured by the SCL-27, was also significantly higher among participants with VBD-c relative to VBD-p (adjusted mean difference 0.18, 95% CI 0.06–0.30; *p* = 0.0034) (Table 4). Perceived stress scores did not differ significantly between subtypes in either unadjusted or adjusted analyses.

**Table 4.** Baseline somatic and psychological symptom burden.

Outcome	Subtype	Mean	Median (IQR)	p-value	Adjusted mean difference (95% CI)	Adjusted p-value
Somatic awareness (PILL)	VBD-c	48.69	46.5 (34–63)			
	VBD-p	35.13	36 (21–47)	<0.0001	14.85 (9.11 to 20.59)	<0.0001
Psychological distress (SCL-27)	VBD-c	0.65	0.52 (0.33–0.90)			
	VBD-p	0.50	0.48 (0.22–0.70)	0.0101	0.18 (0.06 to 0.30)	0.0034
Perceived stress (PSS)	VBD-c	17.58	16 (13–33)			
	VBD-p	16.55	17 (11–22)	0.2696	1.47 (−0.44 to 3.38)	0.2176

### Primary efficacy outcomes

Of the 209 randomized participants, 169 (81%) had complete baseline and Week 16 tampon test pain data and were included in the primary efficacy analyses. Across the four treatment arms, mean reductions in tampon test pain from baseline to Week 16 were modest (Table 5). The largest mean reduction occurred in the combination active treatment arm (oral nortriptyline and lidocaine/estradiol cream; mean change −1.33, SD 2.09). Mean changes in the remaining three arms ranged from −0.66 to −0.69. Median change scores were −1.0 in both lidocaine-containing arms and 0 in both placebo cream arms, reflecting the distribution of change scores within each arm.

**Table 5.** Descriptive change in tampon test pain scores from baseline to week 16 by treatment arm.

Pill	Cream	N	Mean change ± SD	Median change
Nortriptyline	Lidocaine	42	−1.33 ± 2.09	−1.0
Nortriptyline	Placebo	44	−0.68 ± 2.07	0
Placebo	Lidocaine	38	−0.66 ± 1.66	−1.0
Placebo	Placebo	45	−0.69 ± 2.41	0

Change score = week 16 - baseline; negative values indicate reduced pain. Intent-to-treat population.

To assess whether nortriptyline or lidocaine/estradiol was associated with overall improvement in tampon test pain at Week 16, independent of subtype, we ran an ANCOVA model adjusted for baseline pain severity, VBD subtype, and clinic location (Table 6). Baseline tampon test pain was a strong predictor of Week 16 pain (β = 0.67, SE = 0.07; *p* < 0.001), while VBD subtype was not. Neither active nortriptyline versus placebo pill, nor active lidocaine/estradiol versus placebo cream, was associated with a significant reduction in Week 16 pain, and no pill x cream interaction was observed.

**Table 6.** Baseline pain and VBD-subtype adjusted estimates of overall treatment effects on 16-week tampon test pain.

Predictor	β (SE)	95% CI	p-value
Baseline tampon test pain	0.67 (0.07)	0.53 to 0.81	<0.001
Clinic location (UCLA vs. UNC)	0.55 (0.31)	−0.07 to 1.17	0.081
VBD subtype (central vs. peripheral)	0.23 (0.32)	−0.40 to 0.87	0.47
Pill active (nortriptyline vs. placebo)	−0.15 (0.46)	−1.06 to 0.75	0.74
Cream active (lidocaine vs. placebo)	−0.30 (0.45)	−1.18 to 0.58	0.50
Pill × Cream	−0.19 (0.62)	−1.41 to 1.02	0.75

Linear regression model adjusted for baseline pain, VBD subtype, and clinic location in intent-to-treat population. β coefficients represent adjusted mean differences in follow-up pain. Robust standard errors (HC3); R^2^ = 0.40. Intent-to-treat population.

### Treatment effect modification by subtype

We then evaluated whether treatment efficacy differed by VBD subtype using a baseline-adjusted ANCOVA model with subtype–by–treatment interaction terms. A significant interaction emerged between subtype and cream treatment (β = −1.38, 95% CI −2.62 to −0.13; *p* = 0.031), indicating that the effect of lidocaine/estradiol varied by subtype (Table 7). No interaction was observed between subtype and pill treatment. Although participants with VBD-c appeared to experience greater benefit from the lidocaine/estradiol cream than those with VBD-p, the within-subtype effect in VBD-c did not reach statistical significance relative to placebo (*p* = 0.07).

**Table 7.** Subtype-specific estimates of treatment effects on 16-week tampon test pain.

Treatment	Subtype	β (SE)	95% CI	p-value (within-subtype treatment efficacy)	p-value (subtype difference in treatment efficacy)
Nortriptyline pill	VBD-p	−0.18 (0.57)	−1.30 to 0.94	0.75	0.91
Nortriptyline pill	VBD-c	−0.25 (0.54)	−1.32 to 0.82	0.64	
Lidocaine cream	VBD-p	0.46 (0.57)	−0.67 to 1.58	0.42	0.03
Lidocaine cream	VBD-c	−0.92 (0.51)	−1.93 to 0.09	0.07	

Estimates and p-values derived from linear combinations of coefficients from baseline-adjusted subtype–by–treatment interaction model. β coefficients represent mean differences in follow-up pain. Intent-to-treat population.

### Treatment-compliant population

As a secondary supportive analysis, we examined change in tampon test pain among the 103 participants classified as treatment-compliant (Table 8). Treatment allocation was generally balanced across study arms, although the active nortriptyline + lidocaine/estradiol group included slightly more participants than the others (31 versus 24). Overall patterns were consistent with the modified intent-to-treat findings (Table 9).

**Table 8.** Descriptive change in tampon test pain scores from baseline to week 16 in treatment-compliant population.

Pill	Cream	N	Mean change ± SD	Median change
Nortriptyline	Lidocaine	31	−1.45 ± 2.34	−1.0
Nortriptyline	Placebo	24	−0.29 ± 2.29	0.0
Placebo	Lidocaine	24	−0.50 ± 1.69	−1.0
Placebo	Placebo	24	−0.12 ± 2.31	0.0

Change score = week 16 - baseline; negative values indicate reduced pain. Treatment-compliant population.

**Table 9.** Subtype-specific estimates of treatment effects on 16-week tampon test pain in treatment-compliant population.

Treatment	Subtype	β (SE)	95% CI	p-value (within-subtype treatment efficacy)	p-value (subtype difference in treatment efficacy)
Nortriptyline pill	VBD-p	0.05 (0.86)	−1.66 to 1.75	0.9556	0.83
Nortriptyline pill	VBD-c	−0.15 (0.76)	−1.66 to 1.36	0.8434	
Lidocaine cream	VBD-p	0.02 (0.81)	−1.58 to 1.62	0.9834	0.11
Lidocaine cream	VBD-c	−1.41 (0.68)	−2.76 to −0.06	0.0413	

Estimates and p-values derived from linear combinations of coefficients from baseline-adjusted subtype–by–treatment interaction model. β coefficients represent mean differences in follow-up pain. Treatment-compliant population.

Directional Response Patterns. To provide a clinically intuitive view of response patterns, we summarized directional changes in tampon test pain from baseline to Week 16 by treatment arm (Table 10). In both the intent-to-treat and treatment-compliant populations, a substantial proportion of participants in every arm had any decrease, with approximately one-half to two-thirds showing reduced pain over time. Although the combined treatment arm had the highest proportion of participants classified as having any decrease and the double placebo arm had the highest proportion classified as worsened, a decrease was observed across all treatment groups, including placebo-containing arms. Despite these decreases, responses varied considerably within each treatment art, with some participants experiencing substantial reductions in pain and others showing minimal change or symptom worsening (Figure 2). These heterogeneous trajectories align with the primary efficacy findings and underscore the need for future research to identify patient-level factors that predict treatment response. These direction-only categories do not represent a prespecified threshold for clinically meaningful improvement.

**Figure 2.**
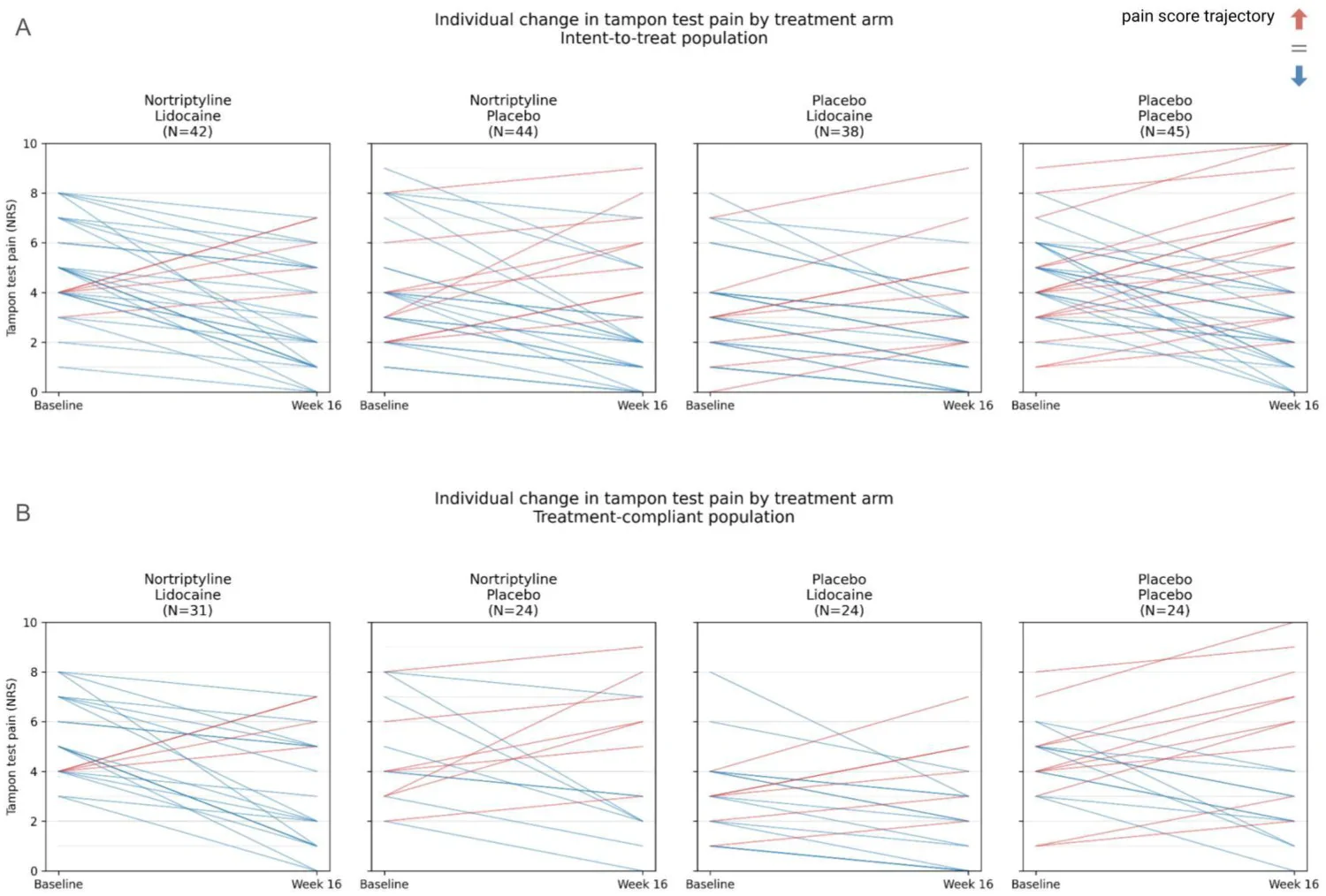
Individual change in tampon test pain by treatment arm. Individual change in tampon test pain by treatment arm. Net directional pain score change by treatment arm for intent-to-treat (A) and treatment-compliant (B) populations. Each line represents an individual participant’s change in tampon test pain score from baseline to Week 16, with red indicating an increase in pain score, grey representing an equivalent pain score, and blue indicating a decreased pain score.

**Table 10.** Direction of change in tampon test pain from baseline to week 16 by treatment arm and analysis population.

Intent-to-treat					
Pill	Cream	N	Any decrease	No change	Worsened
Nortriptyline	Lidocaine	42	26 (61.9%)	11 (26.2%)	5 (11.9%)
Nortriptyline	Placebo	44	21 (47.7%)	14 (31.8%)	9 (20.5%)
Placebo	Lidocaine	38	22 (57.9%)	8 (21.1%)	8 (21.1%)
Placebo	Placebo	45	22 (48.9%)	8 (17.8%)	15 (33.3%)
Treatment-compliant					
Pill	Cream	N	Any decrease	No change	Worsened
Nortriptyline	Lidocaine	31	19 (61.3%)	8 (25.8%)	4 (12.9%)
Nortriptyline	Placebo	24	8 (33.3%)	9 (37.5%)	7 (29.2%)
Placebo	Lidocaine	24	13 (54.2%)	5 (20.8%)	6 (25.0%)
Placebo	Placebo	24	10 (41.7%)	4 (16.7%)	10 (41.7%)

## Discussion

In this randomized, double-blind, placebo-controlled 2 × 2 factorial trial, neither oral nortriptyline (up to 50 mg) nor topical 5% lidocaine/0.5 mg/mL 0.02% estradiol compound cream, administered alone or in combination, produced a statistically significant reduction in tampon test–evoked vulvovaginal pain at 16 weeks compared with placebo in the modified intent-to-treat population. These findings were consistent across analytic approaches, including baseline-adjusted models, and though causally more limited, descriptive analyses restricted to treatment-compliant participants as well.

Despite the absence of statistically significant treatment effects, reductions in tampon test pain were observed across all study arms. Approximately one-half to two-thirds of participants experienced lower tampon-test pain over the study period, with the greatest numerical proportion with any decrease observed in the combined nortriptyline plus lidocaine/estradiol group. Because these differences were not statistically significant, they should be interpreted as descriptive observations rather than evidence of superior treatment efficacy. Nevertheless, the variability in individual responses highlights the heterogeneity of vestibulodynia and suggests that future studies should seek to identify patient characteristics associated with treatment response.

The modest mean improvements observed across all treatment groups, including placebo, are consistent with prior chronic pain trials, including those for VBD [74–78]. Such changes likely reflect a constellation of nonspecific effects, including expectancy, increased clinical attention, regression to the mean, and natural fluctuation in pain and stress over time [79–81]. VBD symptoms are known to vary in intensity and interference [82], in part due to menstrual cycle-related changes [83], and participation in a structured clinical trial may itself influence symptom appraisal. These observations highlight the inherent challenge of distinguishing true pharmacologic effects from contextual influences in chronic pelvic pain research, while also emphasizing that clinical engagement alone may confer benefit.

Baseline comparisons revealed clear differences between VBD-c and VBD-p subtypes, despite similar levels of provoked pain intensity. Participants with VBD-c reported substantially greater pain-related interference with daily functioning, including work and sleep, as well as higher somatic awareness and psychological symptom burden. In contrast, tampon test pain and average vulvar pain severity did not differ between groups. These findings support the hypothesis that VBD subtypes differ primarily in broader symptom experience and central pain processing rather than in localized nociceptive sensitivity. This aligns with existing literature describing heightened central sensitization, altered pain modulation, and greater psychosocial burden among individuals with comorbid chronic pain conditions [10,39,84–87]. Together, these results support further study of subtyping frameworks that incorporate biopsychosocial dimensions rather than relying solely on anatomical localization.

Although lidocaine/estradiol demonstrated no overall efficacy in the full cohort, a secondary cream-by-subtype interaction was observed (*p* = 0.031). Contrary to the a priori hypothesis that VBD-p, presumed to reflect more localized peripheral pathology, would respond preferentially to topical therapy, VBD-c participants showed greater numerical benefit from lidocaine/estradiol, although within-subtype effects did not reach conventional significance (*p* = 0.07), so this result should be considered hypothesis-generating rather than evidence of treatment efficacy.

Several mechanistic explanations may account for this counterintuitive finding. First, peripheral nociceptive sensitivity may differ between subtypes in ways not fully captured by provoked pain testing in the present study. In prior work, we demonstrated that women with VBD-c exhibit greater pelvic muscle pressure pain [10,39], supporting a more nuanced interplay of central and peripheral mechanisms potentially making them paradoxically more responsive to peripheral analgesic interventions. Second, local soothing and anti-inflammatory effects of topical treatment may provide broader benefit than expected, particularly in individuals with heightened central sensitization, where peripheral interventions may be amplified through altered central processing [88] or enhanced placebo responses [89]. Third, hormonal modulation may also play a role. While our data do not provide direct evidence of this and oral contraceptive use was balanced across subtypes, VBD-c responders may have had subtle vestibular tissue changes, including subclinical hormone-mediated atrophy, impaired epithelial barrier function, or localized inflammatory milieu, that rendered them more responsive to hormone exposure [25,26]. These findings challenge dichotomous peripheral-versus-central models and suggest that treatment response reflects complex interactions between tissue factors, central modulation, and psychosocial context.

Directional analyses provided a descriptive complement to model-based results. In both intent-to-treat and treatment-compliant populations, the combined nortriptyline plus lidocaine/estradiol group consistently had the highest proportion of participants experiencing any decrease, whereas the double placebo arm showed the highest proportion with worsening. Substantial heterogeneity in individual response was evident within each treatment arm, with some participants reporting marked reductions in pain, while others experienced minimal change or worsening, a pattern commonly observed in chronic pain trials [90–93]. Although these descriptive analyses do not establish treatment efficacy, they illustrate the marked heterogeneity in individual response and support future studies aimed at identifying patient-level predictors of treatment response.

This trial has several notable strengths. It represents one of the largest randomized controlled evaluations of pharmacologic therapy for VBD, to date, and utilizes a rigorous double-blind, placebo-controlled factorial design. The cohort was extensively phenotyped, enabling characterization of pain subtype, comorbidities, and psychosocial factors. Multiple analytic strategies, including baseline-adjusted models, change-scores analyses, treatment-compliance assessments, and directional trajectories, yielded consistent conclusions. Importantly, the study directly incorporated clinically relevant VBD subtypes that have been underrepresented or excluded from prior trials.

Several limitations warrant consideration. The study enrolled fewer participants than originally planned (target *N* = 400), largely due to COVID-19 pandemic-related disruptions, reducing power to detect modest treatment effects, which limits interpretation of both the null primary findings and the observed subgroup heterogeneity. VBD-p status was based on a single-time-point classification and may be unstable if comorbid conditions emerged later. Compliance was assessed categorically rather than continuously, which may have obscured dose-response patterns. Additionally, intervention-related burden may have influenced adherence, including inconvenience associated with topical cream application and the demands of the oral regimen (up to five study pills daily), as well as potential side effects associated with nortriptyline. Finally, the technical issues which affected collection of SF-MPQ data did not allow us to collect and report these data.

Collectively, these findings underscore the complexity of treating vestibulodynia and the limitations of one-size-fits-all approaches. Although between-arm treatment effects were not statistically significant, substantial heterogeneity in observed pain changes was seen across all treatment arms. Exploratory subtype-specific signals suggest that treatment response may differ across clinically defined groups, though these findings should be considered hypothesis-generating. Pharmacologic therapies may ultimately be most effective when integrated with established nonpharmacologic interventions such as pelvic floor physical therapy and cognitive behavioral therapy [94]. Ongoing analyses will evaluate whether biomarkers can help explain variability in treatment response within this cohort. In parallel, combination and multimodal interventions warrant further investigation, particularly in studies designed to prospectively evaluate mechanistically defined subgroups. Future research should prioritize biomarker-informed stratification and trial designs that can more reliably assess treatment heterogeneity and support individualized approaches to care.

## Conclusions

This large, rigorously conducted factorial trial found that oral nortriptyline and topical lidocaine/estradiol cream, alone or in combination, did not demonstrate statistically significant superiority over placebo for reducing tampon test-evoked pain in women with vestibulodynia. However, reductions in pain were observed across all treatment arms, with 54% of participants having any decrease in pain scores at Week 16. An unexpected secondary cream-by-subtype interaction was observed, suggesting centralized vestibulodynia may show a greater response to lidocaine/estradiol than peripheral vestibulodynia, though the within-subtype lidocaine/estradiol effect in VBD-c was not statistically significant. This challenges simplistic mechanistic models and highlights the importance of phenotypic heterogeneity. Baseline differences between subtypes, characterized by divergent functional impairment and psychosocial burden despite equivalent pain intensity, support further study of clinically-defined subgroups. Although this trial did not identify universally effective pharmacologic interventions, the substantial individual-level heterogeneity in treatment response underscores the critical need for precision medicine approaches integrating mechanistic biomarkers, comprehensive phenotyping, and individualized treatment selection. Future studies should evaluate prediction models that may help match patients to interventions based on underlying pathophysiology.

## Supplementary Material

UPDATe_Supplemental_Table_2 clean(1).docx

UPDATe_Supplemental_Table_1.docx

consort checklist.docx

## Data Availability

De-identified data that support the findings of this study are available from the corresponding author upon reasonable request, subject to institutional review board approval and data use agreements to ensure participant confidentiality.
