# Supplementary material for: Testing peripheral and central therapies for provoked vestibulodynia: results from the UPDATe trial
Source: Ann Med. 2026 Oct 10;58(1):2732293. doi: 10.1080/07853890.2026.2732293 (PMC13656646; doi:10.1080/07853890.2026.2732293)
Supplement: Supplementary file 2 [file IANN_A_2732293_SM2011.docx]

### Supplemental Table 1. Study inclusion and exclusion criteria.

| Inclusion criteria | - Female age 18–50 years - English-literate - BMI under 40 - Willingness to provide informed consent - ≥3 continuous months of pain occurring at the vaginal opening during tampon insertion, penetration, or touch - Average pain ≥3 on a 0–10 numeric rating scale with tampon insertion/removal and/or with insertional sexual activity and/or with touch to the vaginal opening at the first study visit |
| --- | --- |
| Exclusion criteria | - Presence of active dermatologic vulvar disease or vaginal infection - Untreated atrophic vaginitis (participants may undergo treatment with topical hormone therapy for a minimum of six weeks to be considered for reevaluation for enrolment) - Previous vestibulectomy – full or partial - Pregnancy or at risk for pregnancy during the study period - Within the first six months postpartum - Currently breastfeeding/lactating, or within 3 months of discontinuing breastfeeding/lactation - Active incarceration - Cancer diagnosis within the past year - Chemotherapy and/or radiation treatment within the past year - Noncontrolled medical condition: significant renal impairment, hematological disease, hepatic insufficiency, cardiovascular disease (cardiac conduction disturbance, congestive heart failure, hypertension), neurological disorder, autoimmune disease, or respiratory illness - Clear inflammatory states (e.g. Rheumatoid Arthritis, Lupus, Ulcerative Colitis, Crohn’s disease, Ehlers-Danlos Syndrome) - Use of immunosuppressant medications - History of intolerance to nortriptyline, topical lidocaine, or topical estradiol - Contraindications to use of nortriptyline: current use, or use within the past 1-3 months, of MAOIs, some NDRIs, Methylphenidate (DPH), MDPV (Methylenedioxypyrovalerone), Pipradrol (Meratran), Prolintane (Catovit/ Promotil), myocardial infarction in past year, active psychotic or suicidal thoughts, narrow angle closure glaucoma - Discontinuation of medications for 30-90 days, depending on the medication type, dosage, frequency, duration, and half-life. All concomitant medications will need to be documented at the time of screening so the Principal Investigator and medical doctors at each participating site can determine a conservative window. Medications include, but are not limited to: topical lidocaine, estradiol, or lidocaine/estradiol to the vulvar vestibule, nortriptyline or other TCAs, pregabalin, and gabapentin. - Contraindications to the use of lidocaine or local anesthetics - Contraindications to the use of topical estrogen therapy - Post-menopausal status, defined as no menses for 12 consecutive months or surgical removal of both ovaries. (Hysterectomy is not an exclusion) - Botulinum toxin A injections of the pelvic floor muscles in the last 12 months, or pelvic nerve blocks in the last three months - Ongoing pelvic floor physical therapy - Currently enrolled or planning to enroll in another clinical trial during the trial |
