# Supplementary material for: Testing peripheral and central therapies for provoked vestibulodynia: results from the UPDATe trial
Source: Ann Med. 2026 Oct 10;58(1):2732293. doi: 10.1080/07853890.2026.2732293 (PMC13656646; doi:10.1080/07853890.2026.2732293)
Supplement: Supplementary file 3 [file IANN_A_2732293_SM2012.docx]

### Supplemental Table 2. Extended birth control and STD history

| **Characteristic** | **Overall (N=209)** | **VBD-c (N=140)** | **VBD-p (N=69)** | **OR**  **(95% CI)** | **p-value** |
| --- | --- | --- | --- | --- | --- |
| **History of an STD** |  |  |  | 1.45 (0.55 to 3.86) | 0.6073 |
| No | 186 (89.0%) | 123 (87.9%) | 63 (91.3%) |  |  |
| Yes | 23 (11.0%) | 17 (12.1%) | 6 (8.7%) |  |  |
| Type of STD (all that apply) |  |  |  |  |  |
| Chlamydia | 10 (43.5%) | 7 (41.2%) | 3 (50.0%) |  |  |
| Herpes | 8 (34.8%) | 5 (29.4%) | 3 (50.0%) |  |  |
| HPV | 5 (21.7%) | 4 (23.5%) | 1 (16.7%) |  |  |
| Trichomoniasis | 2 (8.7%) | 1 (5.9%) | 1 (16.7%) |  |  |
| Gonorrhea | 2 (8.7%) | 1 (5.9%) | 1 (16.7%) |  |  |
| Other | 2 (8.7%) | 2 (11.8%) | 0 (0.0%) |  |  |
| **Current birth control use** |  |  |  | 1.3 (0.69 to 2.45) | 0.5089 |
| No | 59 (28.2%) | 37 (26.4%) | 22 (31.9%) |  |  |
| Yes | 150 (71.8%) | 103 (73.6%) | 47 (68.1%) |  |  |
| Type of birth control currently used (all that apply) |  |  |  |  |  |
| Birth control pills | 60 (40.0%) | 43 (41.7%) | 17 (36.2%) |  |  |
| Condoms or other barrier methods | 47 (31.3%) | 31 (30.1%) | 16 (34.0%) |  |  |
| Hormonal IUD | 38 (25.3%) | 23 (22.3%) | 15 (31.9%) |  |  |
| Withdrawal or fertility awareness | 24 (16.0%) | 19 (18.4%) | 5 (10.6%) |  |  |
| Abstinence | 13 (8.7%) | 12 (11.7%) | 1 (2.1%) |  |  |
| Other hormonal methods | 17 (11.3%) | 14 (13.6%) | 3 (6.4%) |  |  |
| Copper IUD | 6 (4.0%) | 2 (1.9%) | 4 (8.5%) |  |  |
| Female or male sterilization | 4 (2.7%) | 3 (2.9%) | 1 (2.1%) |  |  |
| Other | 1 (0.7%) | 1 (1.0%) | 0 (0.0%) |  |  |
| **History of hormonal birth control use** |  |  |  | 1.64 (0.88 to 3.05) | 0.1582 |
| No | 61 (29.2%) | 36 (25.7%) | 25 (36.2%) |  |  |
| Yes | 148 (70.8%) | 104 (74.3%) | 44 (63.8%) |  |  |
| **Age when hormonal birth control first used** |  |  |  |  |  |
| 15 or younger | 31 (20.9%) | 20 (19.2%) | 11 (25.0%) |  |  |
| 16 - 20 | 93 (62.8%) | 66 (63.5%) | 27 (61.4%) |  |  |
| 21 -25 | 19 (12.8%) | 14 (13.5%) | 5 (11.4%) |  |  |
| 26 - 30 | 3 (2.0%) | 2 (1.9%) | 1 (2.3%) |  |  |
| 31 - 35 | 2 (1.4%) | 2 (1.9%) | 0 (0.0%) |  |  |
| **Longest period of hormonal contraceptive use** |  |  |  |  |  |
| Less than 1 year | 15 (10.1%) | 9 (8.7%) | 6 (13.6%) |  |  |
| 1 - 3 years | 50 (33.8%) | 41 (39.4%) | 9 (20.5%) |  |  |
| 4 - 5 years | 23 (15.5%) | 14 (13.5%) | 9 (20.5%) |  |  |
| Greater than 5 years | 60 (40.5%) | 40 (38.5%) | 20 (45.5%) |  |  |
